# Morbidity and mortality amongst infants of diabetic mothers admitted into a special care baby unit in Port Harcourt, Nigeria

**DOI:** 10.1186/1824-7288-36-77

**Published:** 2010-12-07

**Authors:** Peace I Opara, Tamunopriye Jaja, Uche C Onubogu

**Affiliations:** 1 Department of Paediatrics, University of Port Harcourt Teaching Hospital, Port Harcourt, Nigeria

## Abstract

**Background:**

Infants born to diabetic women have certain distinctive characteristics, including large size and high morbidity risks. The neonatal mortality rate is over five times that of infants of non diabetic mothers and is higher at all gestational ages and birth weight for gestational age (GA) categories.

The study aimed to determine morbidity and mortality pattern amongst infants of diabetic mothers (IDMS) admitted into the Special Care Baby Unit of University of Port Harcourt Teaching Hospital.

**Methods:**

This was a study of prevalence of morbidity and mortality among IDMs carried out prospectively over a two year period. All IDMs (pregestational and gestational) admitted into the Unit within the period were recruited into the study.

Data on delivery mode, GA, birth weight, other associated morbidities, investigation results, treatment, duration of hospital stay and outcome were collated and compared with those of infants of non diabetic mothers matched for GA and birth weight admitted within the same period. Maternal data were reviewed retrospectively. Data were analyzed using SPSS 16.0.

**Results:**

Sixty percent of the IDMs were born to mothers with gestational diabetes, while 40% were born to mothers with pregestational DM. 38 (74.3%) were born by Caesarian section (CS), of which 20 (52.6%) were by emergency CS. There was no significant difference in emergency CS rates, when compared with controls, but non-IDMs were more likely to be delivered vaginally. The mean GA of IDMs was 37.84 weeks ± 1.88. 29 (61.7%) of them were macrosomic. The commonest morbidities were Hypoglycemia (significantly higher in IDMs than non-IDMs) and hyperbilirubinaemia in 30 (63.8%) and 26 (57.4%) respectively.

There was no difference in morbidity pattern between infants of pre- gestational and gestational diabetic mothers. Mortality rate was not significantly higher in IDMs

**Conclusions:**

The incidence of macrosomia in IDMs was high but high rates of emergency CS was not peculiar to them. Hypoglycaemia and hyperbilirubinaemia were the commonest morbidities in IDMs.

Referring women with unstable metabolic control to specialized centers improves pre- and post- natal outcomes. Maternal-Infant centers for management of diabetes in pregnancy are advocated on a national scale to reduce associated morbidity and mortality

## Background

Diabetes has long been associated with maternal and perinatal morbidity and mortality [1]. Diabetes in Pregnant women could be either pregestational or gestational. Gestational diabetes accounts for about 80% of cases and could represent a predisposition to type 2 diabetes mellitus or an extreme manifestation of metabolic changes during pregnancy [2].

The incidence of impaired glucose tolerance in pregnancy is between 3-10% and varies according to the incidence of diabetes in the general population [3]. In a study in Nigeria; an incidence of 1.7% was recorded with pregestational diabetes accounting for 39% and gestational diabetes for 61% [4].

In an American maternal and infant survey in 1988, 4% of live births were associated with diabetes mellitus, 88% of these were cases of gestational diabetes, 8% were non insulin dependent (type2 DM) and 4% were insulin dependent (type 1 DM) [5].

Perinatal mortality amongst offspring of diabetic mothers has remained high and was previously an indication for termination of pregnancy. The cause of increased perinatal morbidity and mortality is not known but has been attributed to increased insulin levels leading to hyper anabolism. Studies have shown higher mortality amongst infants of diabetic mothers compared to controls [1,4,6]. In the study in Nigeria, the perinatal mortality was found to be 12.5% compared to 3.5% in controls [4]. Perinatal outcome associated with poor glycaemic control in mothers is associated with as high as 49.2% mortality [4].

The major morbidities associated with infants of diabetic mothers include respiratory distress, growth restrictions, polycythaemia, hypoglycaemia, congenital malformations, hypocalcaemia, hypomagnesaemia. However associated macrosomia and increased prevalence of caesarean sections contribute to higher mortality [6,7].

In view of the high morbidity and mortality associated with this condition babies born to diabetic mothers delivered in the University of Port Harcourt Teaching Hospital were studied, to determine common morbidities and need for closer monitoring of these babies.

## Methods

This was a study of prevalence of morbidity and mortality among IDMs, carried out prospectively in the Special Care Baby Unit (SCBU) of the University of Port Harcourt Teaching Hospital between January 2008 and December 2009. Port Harcourt is a cosmopolitan city situated in Rivers State in the southern part of Nigeria. It has a land area of 170 square kilometer with a population of approximately three million, seven hundred and sixty one thousand, six hundred and Forty five. The Hospital is the largest tertiary hospital in the state and serves as a tertiary as well as a general hospital for South- South Nigeria. The SCBU caters for sick newborn infants in the University of Port Harcourt Teaching Hospital and serves as a referral centre for neonates in Port Harcourt and its environs.

All infants of diabetic mothers admitted into the Unit within the period, were consecutively recruited into the study. Informed consent was obtained from the mothers/caregivers and they all gave consent for infant participation in the study. Babies of mothers with pregestational diabetes mellitus (Type 1 and Type 2) and gestational diabetes (GDM) were included. Maternal data were reviewed retrospectively. Information obtained included: a) characteristics of the mothers; age, parity, type and duration of diabetes, treatment received, presence or absence of other illnesses, pregnancy complications, mode of delivery, maternal outcome and b) characteristics of the babies; gestational age, birth weight, diagnosis, results of investigations, treatment received, duration of hospital stay and outcome. For each IDM, an infant of a non diabetic mother admitted into the unit within the same period, matched for gestational age, and birth weight was recruited as control. Sometimes, one infant was used as control for more than one IDM.

Patients were classified into large for gestational age (LGA), appropriate for gestational age (AGA), and small for gestational age (SGA), according to the relationship between intrauterine growth and gestational age [8,9]. Infants whose birth weights were at least 4000 g regardless of gestational age were defined as macrosomic, while those who weighed less than 2500 g were defined as low birth weight.

Investigations done included; random blood glucose, chest radiographs, serum electrolytes, serum bilirubin, complete blood counts and blood cultures. Some of these were done only where applicable. Hypoglycaemia was defined as blood glucose concentrations <2.6 mmol/l, Polycythaemia was defined as a peripheral venous hematocrit greater than 0.65, and hyperbilirubinaemia was defined as serum levels of indirect bilirubin greater than 204 μmol/L (12 mg/dl) and/or any hyperbilirubinaemia requiring treatment (phototherapy and/or exchange blood transfusion). Hypocalcaemia was defined by total serum calcium values lower than 1.50 mmol/L (6 mg/dL). Routine echocardiography could not be done for the babies due to cost and unavailability of the facilities for paediatric echocardiography in the hospital at the time of the study. All babies who were asphyxiated, had significant respiratory distress, or whose mothers were too ill to breastfeed were placed on dextrose infusions until they or their mothers were fit to feed and breast feeding had been well established. All the babies were fed exclusively on breast milk in line with the Hospital's breast feeding policy.

Hypoglycaemia was corrected with 10% dextrose water while hypocalcaemia was corrected with 10% calcium gluconate. All babies with suspected sepsis were placed on broad spectrum antibiotics till blood culture results were reviewed. Data were entered into a Microsoft excel spreadsheet and analysed using SPSS version 16.0. Statistical level of significance was set at p value of 0.05 or less.

## Results

The age range of the mothers of IDMs was 26 - 45 years with a mean of 33.15 ± 4.17 years. All the mothers received antenatal care in the University of Port Harcourt Teaching Hospital either primarily or had been referred from other peripheral hospitals because of a bad obstetric history or because they were diabetics. 31.9% of the mothers of IDMs were primiparous. Eighteen (38.3%) had pregestational DM, while 29 (61.7%) had gestational DM. 31 (66%) had received insulin during pregnancy. Mean blood glucose levels of the mothers as at their last antenatal visit was 11.65 ± 5.19 with a range of 5.0- 22.0 mmol/l. Glycosylated haemoglobin (HbA1 _C_) levels were not routinely measured. Thirty eight mothers (80.8%) delivered via Caesarean section either as emergency (42.5%) or elective (38.3%) while 28 (59.6%) had had previous foetal or neonatal deaths. There was no maternal death. Table 1 shows the characteristics of the mothers.

**Table 1:** characteristics of mothers of infants who were recruited into the study

Characteristic	Number	Percent (%)
**Parity**		
1	15	31.9
2	13	27.7
3	6	12.8
4	5	10.6
≥4	8	17.0
**Total**	**47**	**100**
**Type of diabetes**		
GDM	29	61.7
Type 1	10	21.3
Type 2	8	17.0
**Total**	**47**	**100**
**Previous foetal/neonatal deaths**		
Foetal	20	42.5
Neonatal	8	17.1
None	19	40.4
**Total**	**47**	**100**
**Mode of delivery**		
Elective CS	18	38.3
Emergency CS	20	42.5
Spontaneous vaginal	7	14.9
Assisted vaginal (vacuum)	2	4.3
**Total**	**47**	**100**

Of the IDMs, 31 were males and 16 were females with a M: F ratio of 1.9:1. Gestational ages ranged from 31 - 41 weeks with a mean of 37.84 ± 1.88 weeks. The mean birth weight was 4.14 kg ± 0.838 (2.0 - 6.0 kg). 29 (61.7%) were macrosomic. Mean Apgar scores at 1 minute was 6.96 ± 1.62; and at 5 minutes, 8.45 ± 1.16. 91.5% of the babies were admitted into the ward within the first hour of birth. Duration of admission was 1-21 days with a mean of 6.97 ± 2.63 days.

Table 2 shows the general characteristics of the IDMs and duration of admission.

**Table 2:** Characteristics of infants of diabetic mothers

Characteristics	Number	Percent (%)
**Gestational age (weeks)**		
<37	7	14.9
37-41	37	78.7
≥41	3	6.4
**Total**	**47**	**100**
**Birth weight (kg)**		
<2.5	4	8.5
2.5-3.99	14	29.8
≥4	29	61.7
**Total**	**47**	**100**
**Feeding commenced (hours)**		
<24	14	29.8
24-72	19	40.4
>72	14	29.8
**Total**	**47**	**100**
**Duration of admission (days)**		
≤3	11	23.4
4-7	22	46.8
≥7	14	29.8
**Total**	**47**	**100**

The commonest morbidities were hypoglycaemia 30 (63.8%), neonatal jaundice 27 (57.4%), and respiratory disorders 16 (34.0%). Mean blood glucose levels of all IDMs within the first hour was 2.93 ± 1.51 mmol/l. Thirty (63.8%) developed hypoglycaemia within the first 24 hours.

Of all the IDMs with neonatal jaundice, 14 were macrosomic and 4 (14.8%) required exchange blood transfusion. There was no statistically significant difference in the occurrence of jaundice between macrosomic and non macrosomic babies (p = 0.49). Respiratory distress was noted in 16 (34.0%). There was radiological evidence of transient tachypnoea of the new born in 10 (62.5%), congenital pneumonia in 5 (31.3%) and respiratory distress syndrome in 1 (6.3%).

Fifteen babies (31.9%) had features suggestive of sepsis but only 3 (0.2%) had culture proven sepsis. Mean PCV levels were 54.5% ± 7.311 (43 -69%). 5 (10.6%) had polycythaemia, of which one had a partial exchange blood transfusion. Hypocalcaemia occurred in 11 (23.4%) babies. Table 3 shows the morbidities and treatment received.

**Table 3:** Morbidities and treatment received

Diagnoses	Number	Percent (%)
Hypoglycaemia	30	63.8
Neonatal jaundice	27	57.4
Respiratory distress	16	34.0
Neonatal sepsis	15	31.9
Birth asphyxia	7	14.9
Polycythaemia	5	10.6
Birth injuries	2	4.3
Seizures	1	2.1
Hypocalcaemia	11	23.4
Hypomagnesemia	5	10.6
Hypophosphatemia	4	8.5
Others	2	4.3
**Treatment**		
Phototherapy	30	63.8
Dextrose infusions	47	100
Antibiotics	35	68.1
Oxygen therapy	7	14.9
Calcium supplements	11	23.4
Exchange blood transfusion	5	10.6

Mortality was recorded in 2 (4.3%) of the babies. The babies who died were macrosomic and severely asphyxiated, and were delivered via emergency caesarean section following prolonged obstructed labour and late presentation to hospital.

There was no significant difference in the morbidity pattern between infants of pregestational and gestational diabetic mothers. Table 4 shows the clinical and biochemical characteristics of gestational and pregestational diabetic mothers/infants.

**Table 4:** Clinical and biochemical characteristics of pregestational and gestational diabetic mothers/infants

	Pregestaional DM	Gestational DM	p value
	N = 18	N = 29	
**Mode of delivery**			
EMCS	8	12	0.895
ELCS	6	12	0.71
SVD	4	4	0.53
**Birth weight (kg)**			
<2.5	2	2	0.64
2.5 - 3.99	7	9	0.70
≥4.0	9	18	0.67
**Hypoglycaemia**	8	18	0.52
**Normoglycaemia**	10	11	0.47
**Jaundice**	11	14	0.63

There were 22 controls with gestational ages ranging from 32 - 41 weeks (38.8 ± 1.424) and a M:F ratio of 1: 1.5. Their mean birth weight was 4.37 ± 0.772, (2.3 -6.0 kg), mean apgar scores at one minute were 5.95 ± 2.12 and at 5 minutes, 7.80 ± 2.093. Thirteen were macrosomic.

Table 5 shows a comparison of some parameters between IDMs and non- IDMs. Emergency caesarian section rates were also high in the non-IDMs, but they were more likely to be delivered vaginally. The commonest morbidities among the controls were birth asphyxia, neonatal Sepsis/infections and respiratory distress. Transient tachypnoea of the newborn and pneumonia accounted for all the cases of respiratory distress. There was no case of respiratory distress syndrome or polycythaemia. There was no mortality recorded in the control group.

**Table 5:** Comparison of delivery mode and common morbidities between IDMs and non-IDMs

	IDMS	Non-IDMs	P value
	N = 47	N = 22	
**Delivery mode**			
EMCS	20	10	0.926
ELCS	18	2	0.098
SVD	4	10	0.013
**Common Morbidities**			
Hypoglycemia	26	3	0.048
Neonatal jaundice	25	4	0.109
Neonatal sepsis/infections	16	8	0.902
Respiratory distress	16	8	0.902
Birth asphyxia	7	8	0.202
Birth injuries	2	3	0.328
Mortality	2	0	1.000

## Discussion

This study shows that 28 (59.6%) of the diabetic mothers had had previous foetal or early neonatal deaths, this being one of the reasons (as documented in their case notes) for referral of some of them to our centre. This shows that diabetes still contributes significantly to perinatal and neonatal mortality in our environment. The study also showed a low mortality rate among IDMs, although still higher than in the non- IDMs. Although perinatal mortality among this group has declined [10,11], neonatal morbidity remains a significant challenge [12-14].

There was a high incidence of macrosomia among IDMs in this study. This finding is similar to that in another Nigerian study [5]. Macrosomia remains an important morbidity because it is associated with increased risk for traumatic birth injury, obesity, and diabetes in later life [12]. Although some of the variation in incidence may be related to definition, most authors agree that macrosomia is in part related to maternal glucose control [15,16].

Glycaemic control in our mothers was difficult to ascertain since only blood glucose levels done during antenatal visits were documented and glycosylated haemoglobin (HbA1_C_) levels were not routinely measured. In centers that have coordinated diabetes-in-pregnancy programmes which stress good maternal glycaemic control, the incidence of macrosomia is reduced [17]. The high rates of macrosomia in this study may therefore reflect poor glycaemic control in the mothers.

The caesarian section (CS) rate in this study was very high with emergency CS rates being higher than elective CS rates even amongst the controls. The high rate of operative deliveries, similar to the other Nigerian study [5] is related to the high incidence of macrosomia in the IDMs and their matched controls. Our findings were however in contrast to the findings in a Sri-Lankan study [18] where elective CS rates were much higher than emergencies. Sri Lanka holds a unique place in South Asia as one of the first of the less developed nations to provide universal health [19]. In our environment women usually register late for ANC and the acceptability of operative deliveries is very low [5,20-22].

Thus, a woman booked for an elective CS will often show up when an emergency CS is inevitable.

Hypoglycaemia was the commonest neonatal problem recorded in the IDMs, occurring in significantly higher proportions than in the non-IDMs. The high rate of hypoglycaemia in our IDMs is similar to findings by other authors [13,14,18] and has been identified as a marker of poor glycaemic control in the mother. A rapid decline in plasma glucose concentration is characteristic of the IDM, especially one whose mother's diabetes is poorly controlled [10,13]. Maternal hyperglycemia leads to foetal hyperglycaemia, stimulating the foetal pancreas to synthesize excessive insulin. With separation of the placenta at birth, there is a sudden interruption of glucose infusion to the neonate without a proportional effect on hyperinsulinemia [10,13]. Thus hypoglycaemia develops within the first hours of birth. Although there were no proper records of peripartum glucose levels in our mothers, mean blood glucose levels were high and the high rates of neonatal hypoglycaemia may also be a reflection of poor maternal glycaemic control.

Hypocalcemia was the other common metabolic problem in this study. It has also been documented as a problem of IDMs by other authours [13,14,23,24]. Tsang et al [25] advanced the hypothesis that hyperparathyroidism of diabetic mothers might suppress the foetal parathyroid function and lead to hypocalcaemia of the newborn.

Neonatal jaundice was noted very frequently in our IDMs, irrespective of the type of maternal diabetes. Its incidence was however not significantly higher than in the control group, probably because these were matched controls who had been admitted for other illnesses, like jaundice from blood group incompartibilities and also probably because of our small sample size. The high rates of jaundice in our IDMs was in contrast with another African study where it occurred in only 29% of cases. The reason for this contrast is not clear. Hyperbilirubinaemia is a recognised problem of infants of diabetic mothers, and has been shown to occur with increased frequency in macrosomic infants of diabetic mothers [26,27]. Hyperbilirubinemia occurred in about half of our macrosomic IDMs although this association was not statistically significant.

Congenital pneumonia and transient tachypnoea of the newborn (TTN) were common causes of respiratory distress in all the babies this study. This is not surprising as infections, to which IDMs are also at increased risk play a major role in neonatal morbidity and mortality in our environment [28]. TTN is a known complication of IDMs especially following caesarean section deliveries [23]. There was also a high rate of TTN among the controls, which was expected because most of them were also macrosomic and delivered by caesarian section, which on its own is a known risk factor.

TTN was also common in a similar study done in Harare [24]. However studies done outside Africa [13,18] recorded respiratory distress syndrome as the commonest cause of respiratory distress amongst IDMs. The Harare study [24] did not record any case of respiratory distress syndrome while we recorded only one case and none among the non-IDMs. This may buttress the fact that although RDS is one of the commonest causes of respiratory distress in other races, it is uncommon in Africa [29].

There were no obvious congenital malformations in our study. The incidence of major congenital malformations has been reported to be 2-5 times greater in IDMs than in other infants with cardiac malformations recorded as the most common [30,31]. The unavailability of routine echocardiography in this study may have missed out cases of asymptomatic congenital cardiac malformations. The incidence was also high in the other Nigerian study [5]. This may also be because they studied outcome in pregnant women whilst we studied only babies admitted into our unit, thereby also possibly excluding malformations which were not compatible with life. The Harare study [24] however also recorded a low incidence of life threatening congenital malformations.

The study did not show any significant difference in morbidity pattern between infants of mothers with pregestational diabetes and those with gestational diabetes. This is a pointer to the need for screening of all pregnant women for diabetes and also anticipatory and active management of infants of diabetic mothers irrespective of type of maternal diabetes. The mortality rate was lower among the non IDMs but the difference was not statistically significant. This shows that with proper care, live born IDMs without major congenital malformations have a good chance of survival.

## Conclusions

The study shows a high incidence of macrosomia, hypoglycaemia and hyperbilirubinaemia but absence of obvious congenital abnormalities in IDMs. They were more likely to be delivered by Caesarian sections than non- IDMs but rates of emergency CS among the two groups were similar. Morbidity pattern was identical in both infants of pregestational and gestational diabetes mellitus.

Screening of all pregnant women for diabetes, good glycaemic control and active management of their infants will reduce perinatal morbidity and mortality. Opening of maternal-infant centres with standard protocols for prevention and treatment of diabetes in pregnancy on a national scale will go a long way in reducing the scourge of this condition.

## Competing interests

The authors declare that they have no competing interests.

## Authors' contributions

PIO, UCO contributed to conception, design and acquisition of data.

PIO, TJ did the analysis and interpretation of data and were involved in drafting the manuscript.

All authors participated in reading and revising the manuscript and gave final approval of the version to be submitted.
